# Plant Viral Nanoparticle Conjugated with Anti-PD-1 Peptide for Ovarian Cancer Immunotherapy

**DOI:** 10.3390/ijms22189733

**Published:** 2021-09-08

**Authors:** Aayushma Gautam, Veronique Beiss, Chao Wang, Lu Wang, Nicole F. Steinmetz

**Affiliations:** 1 Department of NanoEngineering, University of California, San Diego, CA 92093, USA; agautam@ucsd.edu (A.G.); vbeiss@eng.ucsd.edu (V.B.); chaow0704@gmail.com (C.W.); 2 Department of Bioengineering, University of California, San Diego, CA 92093, USA; luw008@eng.ucsd.edu; 3 Department of Radiology, University of California, San Diego, CA 92093, USA; 4 Center for Nano-ImmunoEngineering, University of California, San Diego, CA 92093, USA; 5 Moores Cancer Center, University of California, San Diego, CA 92093, USA; 6 Institute for Materials Discovery and Design, University of California, San Diego, CA 92093, USA

**Keywords:** cowpea mosaic virus, checkpoint inhibitor therapy, anti-PD-1 blockade, in situ vaccine, cancer immunotherapy

## Abstract

Immunotherapy holds tremendous potential in cancer therapy, in particular, when treatment regimens are combined to achieve synergy between pathways along the cancer immunity cycle. In previous works, we demonstrated that in situ vaccination with the plant virus cowpea mosaic virus (CPMV) activates and recruits innate immune cells, therefore reprogramming the immunosuppressive tumor microenvironment toward an immune-activated state, leading to potent anti-tumor immunity in tumor mouse models and canine patients. CPMV therapy also increases the expression of checkpoint regulators on effector T cells in the tumor microenvironment, such as PD-1/PD-L1, and we demonstrated that combination with immune checkpoint therapy improves therapeutic outcomes further. In the present work, we tested the hypothesis that CPMV could be combined with anti-PD-1 peptides to replace expensive antibody therapies. Specifically, we set out to test whether a multivalent display of anti-PD-1 peptides (SNTSESF) would enhance efficacy over a combination of CPMV and soluble peptide. Efficacy of the approaches were tested using a syngeneic mouse model of intraperitoneal ovarian cancer. CPMV combination with anti-PD-1 peptides (SNTSESF) resulted in increased efficacy; however, increased potency against metastatic ovarian cancer was only observed when SNTSESF was conjugated to CPMV, and not added as a free peptide. This can be explained by the differences in the in vivo fates of the nanoparticle formulation vs. the free peptide; the larger nanoparticles are expected to exhibit prolonged tumor residence and favorable intratumoral distribution. Our study provides new design principles for plant virus-based in situ vaccination strategies.

## 1. Introduction

The immune system plays a critical role in tumor surveillance, and cancer immunotherapy is a recognized pillar of cancer therapy. Aggressive tumors present with an immunosuppressed tumor microenvironment (TME), which hinders intrinsic anti-tumor immunity [1]. Immunotherapies targeting the TME and the various checkpoints of the cancer immunity cycle hold promise in cancer immunotherapy because these approaches modulate the activity of the immune system to promote its anti-tumor functions [2]. Various immune checkpoints have been identified, with the PD-1/PD-L1 pathway being a prominent target for cancer immunotherapy and several approved monoclonal antibody therapies, such as Nivolumab by Bristol-Myers Squibb and Pembrolizumab by Merck. The PD-1 (programmed cell death-1) receptor is expressed on T cells and its ligand, PD-L1, on innate immune cells including macrophages as well as on cancer cells. PD-1 and PD-L1 are co-inhibitory factors that function as a “brake” to keep immune responses under control. Immune checkpoint inhibitors that target the PD-1/PD-L1 axis overcome inhibition of effector T cell function, in other words, PD-1/PD-L1 inhibitors take the “brakes” off T cells, enabling tumor cell killing. Therapeutics such as Nivolumab and Pembrolizumab show promise as mono- and combination therapy. While an immune checkpoint blockade has produced remarkable clinical outcomes for patients [3], most patients do not respond optimally, or they develop resistance [4]. Strategic immuno-combination therapies are the formula for success; more than 800 registered oncology trials have focused on combination therapies. 

A particularly powerful approach is the combination of in situ vaccination strategies with immune checkpoint blockade. The in situ vaccination strategies makes use of immunostimulatory agents administered directly into an identified tumor; the immunostimulatory agent acts as adjuvant to recruit and activate innate immune cells, and the patient’s tumor provides the source of antigen. Immune cell mediated tumor cell death releases tumor-associated antigens (TAAs) for processing by the innate immune cells, which become antigen-presenting cells (APCs), to then prime the adaptive arm, leading to systemic anti-tumor immunity and memory [5].

Several in situ vaccine approaches are in preclinical and clinical development and therapies such as Imlygic (Amgen) are already in clinical use. Imlygic is an engineered oncolytic virus administered intralesionally; tumor cell killing is achieved by the oncolytic function of the virus, which is engineered to express the cytokine granulocyte-macrophage colony-stimulating factor (GM-CSF), to recruit and activate innate immune cells, to process TAAs, and that lead to systemic anti-tumor immunity [6]. As an alternative to the mammalian viruses, our group has focused on the development and study of plant viral immunotherapies. We have demonstrated the immunomodulatory properties of the plant virus cowpea mosaic virus (CPMV).

CPMV is an icosahedral plant virus measuring 30 nm in diameter; the virus particles are non-enveloped and non-glycosylated. Although CPMV is non-infectious toward mammals, it is immunogenic. The repetitive, multivalent protein assemblies are pathogen-associated molecular patterns (PAMPs) that act as danger signals and activate the innate immune system. The primary PAMP receptors that recognize proteins are Toll-like receptors (TLRs). Our data indicate that RNA-free, empty CPMV (eCPMV) particles are recognized by TLR2 and TLR4. RNA-containing CPMV particles signal additionally through TLR7 [7]. In particular, CPMV signals through interferon gamma (IFN-γ) [8,9] as well as type-I interferons [7]. The innate immunostimulation recruits innate immune cells into the TME, i.e., reprogramming of M2 to M1 macrophages, infiltration of N1 neutrophils, etc. Tumor cell killing is initially mediated by neutrophils and natural killer (NK) cells; activation and recruitment of antigen presenting cells then lead to priming of systemic anti-tumor immunity. We demonstrate priming of tumor-specific CD4^+^ and CD8^+^ cells, including memory cells; the adaptive arm targets metastatic disease and induces immune memory [10]. We have demonstrated that CPMV in situ vaccination stimulates a potent antitumor immune response in mouse models of melanoma, ovarian cancer, breast cancer, colon cancer [8,9], and glioma [11]. CPMV induces systemic and durable immune-mediated anti-tumor efficacy accompanied with immunological memory to prevent recurrence [9]. Ongoing trials in companion dogs with melanoma indicate that the potent antitumor efficacy of CPMV can be replicated in these patients [12,13,14]. 

CPMV in situ vaccination primes innate immune cell activation, which leads to adaptive immune system-mediated, anti-tumor responses. These responses include increased tumor infiltration by CD4^+^ and CD8^+^ effector T cells and memory T cells. Following CPMV in situ vaccination, expression of PD-1 and PD-L1 is differentially increased in tumor models of melanoma, ovarian carcinoma, and colon carcinoma. Therefore, CPMV treatment sensitizes the tumor to a specific immune checkpoint therapy and combination therapy, showing dramatic increases in efficacy against tumors such as ovarian cancer [15]. 

Building on this prior work, we set out to develop a next-generation CPMV displaying anti-PD-1 peptides. Small molecule agents and peptides have been developed to target the PD-1/PD-L1 axis. For example, the macrocyclic peptide BMS-986189 is undergoing clinical testing in a phase I clinical trial (NCT02739373). Another candidate is the D-peptide ^D^PPA-1 (NYSKPTDRQYHF), which blocks PD-1–PD-L1 interactions. The D configurations confers stability, and in vivo efficacy was demonstrated in tumor mouse models [16]. Another peptide in development is AUNP-12, a branched peptide with SNTSESF- branched off the main sequence SNTSESFKFRVTQLAPKAQIKE at the K residue (underlined). The peptide was designed to mimic the endogenous PD-1 receptor and inhibits PD-1 function; in particular, the side branch was shown to have surprisingly high activities [17]. In this work, we chose the side branch SNTSESF (also known as AUR-7) and conjugated it to CPMV nanoparticles to test the hypothesis that CPMV displaying the anti-PD-1 peptide would show enhanced efficacy as an in situ vaccine. We report the bioconjugation of CPMV-SNTSESF, referred to as CPMV-AUNP, and demonstrate efficacy using a mouse model of metastatic ovarian cancer.

## 2. Results

### 2.1. Preparation and Characterization of the CPMV-AUNP Formulation

**Figure 1:**
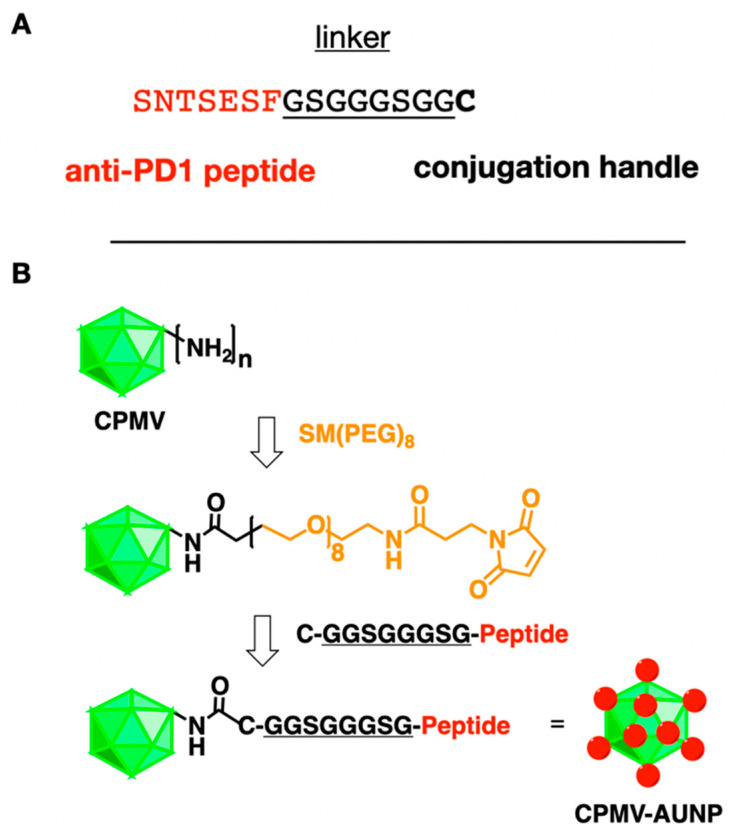
**Conjugation scheme.** (**A**) Amino acid sequence of the anti-PD-1 peptide SNTSESF and its linker and carboxy-terminal cysteine reside. (**B**) Bioconjugation scheme showing CPMV and its solvent-exposed amine groups from lysine side chains, followed by conjugation of the SM-(PEG)_8_ linker, introducing a maleimide group that then reacts with the cysteine side chain of the peptide SNTSESFGSGGGSGGC (the terminal C is shown in bold in the structure).

CPMV-AUNP was obtained by conjugating SNTSESF via an intervening linker GSGGGSGG and carboxy-terminal cysteine residue to CPMV using an AUR-7 peptide [17] SNTSESFGSGGGSGGC to CPMV using a bi-functional *N*-hydroxysuccinimide (NHS)-PEG_8_-maleimide (SM-PEG_8_) linker (Figure 1). CPMV nanoparticles present 300 addressable, solvent-exposed lysine side chains for functionalization [18]. First, SM-PEG_8_ is added to CPMV whereby the NHS arm of the SM-PEG_8_ linker connects to CPMV’s surface lysines to form a stable amide bond, exposing the functional maleimide handle for further bioconjugation. Then the peptide is added, and the maleimide-functional group displayed on CPMV-SMPEG allows conjugation of the cysteine-terminated anti-PD-1 peptide SNTSESFGSGGGSGGC. The resulting CPMV-AUNP is then purified by spin filtration using 100 kDa cut off centrifugal devices to remove excess peptides and is then characterized using a combination of native and denaturing gel electrophoresis, transmission electron microscopy (TEM), size exclusion chromatography (SEC) to confirm structural integrity of the nanoparticles and to determine the number of peptides per CPMV-AUNP. 

**Figure 2:**
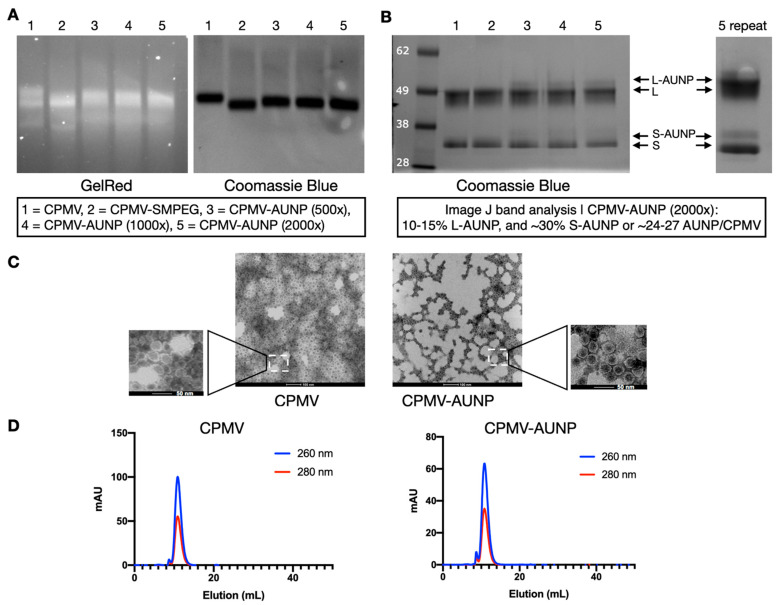
Characterization of CPMV-AUNP. (**A**) Agarose gel electrophoresis of CPMV, CPMV-SMPEG, and CPMV-AUNP stained for GelRed (RNA detection) and Coomassie Blue (protein detection) and imaged under UV and white light, respectively. (**B**) SDS-PAGE analysis of the denatured coat proteins, S and L of CPMV, as well as the AUNP-conjugated versions thereof. S and L proteins have a molecular weight of 24 kDa and 42 kDa, respectively, and SNTSESFGSGGGSGGC has a molecular weight of 1.3 kDa. The left lane shows the molecular weight of the See Blue Plus 2 protein marker. (**C**) TEM of negatively stained CPMV and CPMV-AUNP. The scale bars are 100 and 50 nm in the insets. (**D**) SEC using a Superose-6 increase column on the ÄKTA Explorer system; RNA is monitored using a 260 nm and protein is monitored at 280 nm detector.

First, CPMV vs. CPMV-SMPEG vs. CPMV-AUNP formulations were analyzed by native agarose gel electrophoresis (Figure 2A), and after electrophoretic separation, gels were stained with GelRed and imaged under UV light to detect the encapsidated nucleic acids and then stained with Coomassie Brilliant Blue and imaged under white light to detect the protein capsids. Native agarose gel electrophoresis indicated that all samples analyzed remained intact: the RNA and protein bands co-localized and there was no evidence of broken particles, free RNA or aggregation. The CPMV-SMPEG formulation had higher electrophoretic mobility toward the anode, which was in agreement with positive charge reduction as a neutral linker (SM-PEG_8_) conjugated to surface lysines. Mobility of CPMV-AUNP conjugates was reduced compared to the CPMV-SMPEG formulation, which could be explained by an interplay of charge and increased molecular weight. SNTSESFGSGGGSGGC has a molecular weight of 1.4 kDa and pI of 3.3 and a net negative charge of −1 (peptide calculator, https://www.bachem.com/, accessed on 11 August 2021). We tested conjugation of SNTSESFGSGGGSGGC to CPMV using an excess of 500, 1000, and 2000 M peptide to CPMV (Figure 2A, lanes 3–5). Data indicated increasing mobility toward the anode with increased molecular excess used, which might have indicated that the higher molar excess used led to increased peptide conjugation, therefore increasing the negative charge of CPMV-AUNP and its mobility toward the anode. Second, SDS-PAGE confirmed the covalent attachment of the anti-PD-1 peptide to CPMV. In addition to the small (S, 24 kDa) and large (L, 42 kDa) CPMV coat proteins, higher molecular weight bands were detected for the CPMV-AUNP formulations (Figure 2B). Band analysis using ImageJ software (version 1.53a) indicated that 24–27 AUNP were conjugated to CPMV when using a 2000 M excess of peptide per CPMV. Data were reproducible under these bioconjugate reaction conditions; therefore, we chose the 2000 M excess for any experiments going forward.

To verify the structural integrity of CPMV-AUNP, TEM imaging of negatively-stained samples was conducted, and imaging confirmed the presence of intact, monodisperse nanoparticles measuring 30 nm in size (Figure 2C). There was no apparent difference comparing CPMV to CPMV-AUNP. This was further validated by SEC, which indicated intact CPMV and CPMV-AUNP eluting from the Superose 6 increase column; RNA (detected at 260 nm) and protein (detected as 280 nm) co-elute at ~12 mL with A260:280 nm ratio of ~1.8, which indicates presence of intact CPMV and CPMV-AUNP particles (Figure 2D). Broken or disassembled coat protein units or particle aggregation was not apparent from SEC measurements, and this is consistent with agarose gels and TEM data.

### 2.2. In Vivo Efficacy of the CPMV-AUNP Formulation against Ovarian Cancer

To assay efficacy of the CPMV-AUNP nanoparticle, we used a syngeneic mouse model of serous ovarian cancer. Specifically, we used hyper-aggressive ID8defb29/vegf cells administered intraperitoneally (IP) in C57BL/6J mice. We chose this mouse model because the histopathology and immunological response of these tumors closely resembles human disease [19]. Only female animals were used because we were targeting ovarian cancer. We used luciferase-labeled ID8defb29/vegf cells, allowing us to quantify the disease burden and establishment of disease (data not shown). Imaging is only informative at early time points, before the development of ascites; therefore, we used imaging to confirm successful tumor cell injection and tumor formation but then monitored body weight to assess tumor burden, and 35 grams body weight was defined as the endpoint for the study. After tumor inoculation, mice were randomly assigned into treatment groups and treatment was given by IP administration on day 8, 15, 22, 29, 36, and 42. The following treatment arms were assigned (200 µL IP injection): PBS control (*n* = 5), 1 µg AUNP (*n* = 3), 100 µg CPMV (*n* = 5), 100 µg CPMV-AUNP (*n* = 7), 100 µg CPMV+ 1 µg AUNP physical mixture (*n* = 7).

**Figure 3:**
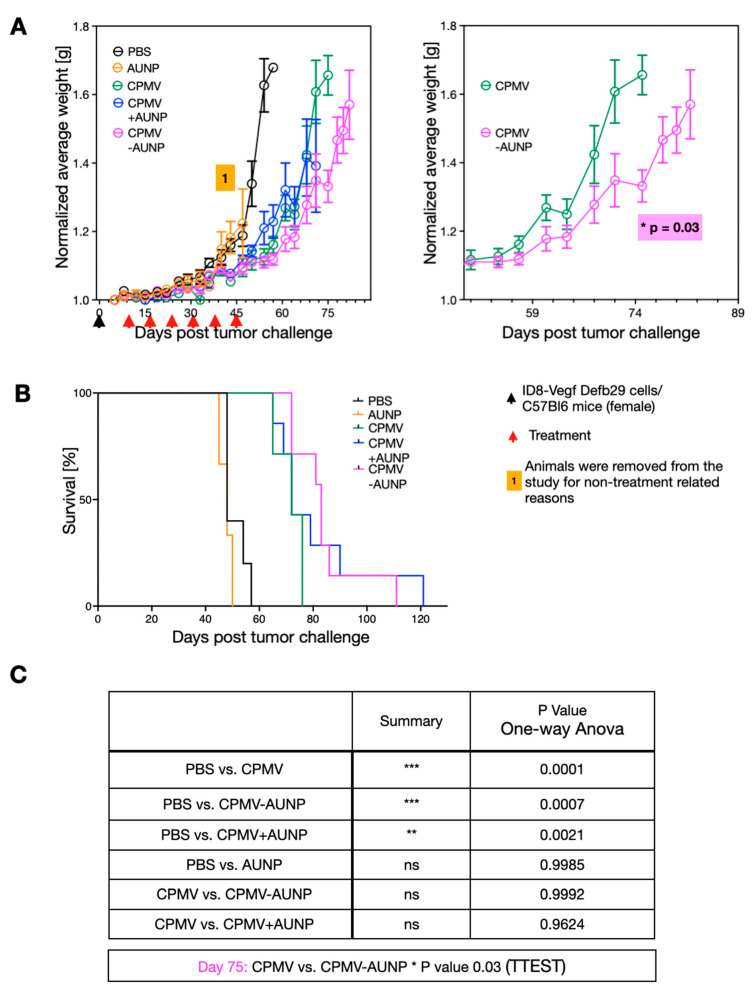
CPMV-AUNP as cancer immunotherapy against ovarian cancer. (**A**) Female C57BL/6 mice were inoculated (i.p.) with 2 × 10^6^ ID8-Defb29/Vegf-A cells, followed by six weekly injections (i.p.) of PBS control (*n* = 5), 1 µg AUNP (*n* = 3), 100 µg CPMV (*n* = 5), 100 µg CPMV-AUNP (*n* = 7), and 100 µg CPMV+ 1 µg AUNP physical mixture (*n* = 7). Body weight was measured to monitor tumor growth. Right panel excerpt of complete data set from the left panel. Data are means ± SEM. Data are plotted for a minimum of *n* = 3 per group. (**B**) Survival curves of the treatment groups. (**C**) Statistics of data presented in panel A; statistical significance was calculated by one-way ANOVA and t test. *** *p* < 0.001, ** *p* < 0.005, ns: not significant.

All control animals (PBS-treated) reached endpoint at day 57 (Figure 3A,B). As previously demonstrated [10], weekly treatment using CPMV at a dose of 100 µg showed efficacy against these aggressive and disseminated ovarian tumors, resulting in prolonged survival: four out of six animals reached endpoint at day 71 and two animals reached endpoint at day 75 (Figure 3A,B). Free AUNP peptide had no apparent effect at the weekly dosing using 1 µg (this dose was matched to the dose of peptide administered when conjugated to CPMV-AUNP). Animals in this group had to be removed from the study due to non-treatment related reasons at day 47. However, up to this day, the tumor burden as measured by increase in body weight matched closely with the PBS control group, indicating that at this dose, the minimal sequence of SNTSESF was not effective as a solo-treatment arm (Figure 3A,B). Similar data indicate that the physical mixture of CPMV and the AUNP peptide did not improve efficacy beyond that observed for CPMV alone. In contrast, moderately increased efficacy was apparent for the CPMV-AUNP group: only one animal reached endpoint at day 75 (all CPMV animals reached endpoint at this timepoint); two animals in the CPMV-AUNP remained in the study until day 85 with the last animal reaching endpoint at day 99 (Figure 3A,B). Divergence of the tumor growth curves (measured based on body weight increase due to ascites formation) was apparent from day 45, with statistical significance observed from day 75 (Figure 3A, right panel; Figure 3C). While the increase in tumor efficacy was moderate, the minimal peptide SNTSESF conjugated at a sparse density (<30 copies per 30 nm-sized nanoparticle), increasing the potency of CPMV alone. This was only observed for the conjugated formulation and not when SNTSESF was added to CPMV as free peptide. This could be explained by the differences in the in vivo fates of the nanoparticle formulation vs. the free peptide, the latter of which likely experienced rapid wash out effects from the tumor, while the larger nanoparticles were expected to exhibit prolonged tumor residence and altered intratumoral distribution. 

## 3. Discussion

We report the design of CPMV-AUNP nanoparticles and demonstrate efficacy against tumors in a mouse model of disseminated and aggressive ovarian cancer. Ovarian cancer is the foremost cause of gynecological cancer and a major cause of cancer death in women [20]. A clinical challenge is that the disease is often not diagnosed prior to stage III (metastasis to peritoneal cavity) or stage IV (metastasis outside of peritoneal cavity); thus, most patients present with a highly metastatic disease that cannot be cured surgically. Surgical debulking followed by chemotherapy is the standard of care. Relapse occurs in 70–90% of stage III and 90–95% of stage IV [21]. Immunotherapy is now established as the fourth pillar of cancer treatment, and for certain cancers, has already significantly reduced mortality [22,23,24]. Immunotherapies such as in situ vaccination approaches as we described here hold tremendous potential to improve patient outcomes and save lives of women with ovarian tumors.

We previously reported efficacy of the CPMV in situ vaccine when used as solo therapy as well as in combination with chemotherapy [25], radiation [26] and immune checkpoint therapy [15]. With regards to CPMV and immune checkpoint therapy combinations, this is a particularly powerful approach: in situ vaccination with CPMV increases antigen specific effector T cells, and this is how it generates systemic resistance to the treated tumor. To be effective, such a vaccine fueled immune activation also requires release of the immunosuppressive “brakes” (e.g., PD-1/PD-L1) that are upregulated in aggressive tumors and in response to the immunotherapy. Immune checkpoint blocking antibodies are effective at removing inhibitory signals but as monotherapy is efficient only against highly immunogenic tumors, expanded efficacy in non-immunogenic tumors can be achieved through immunogenic interventions such as in situ vaccines to enable a tumor antigen-specific cytotoxic T lymphocyte response. We have shown that combination of CPMV with anti-PD-1 antibodies results in improved anti-tumor efficacy in tumor mouse models [15]. 

Here, we extended this work and combined CPMV with an anti-PD-1 peptide; specifically, we chose the minimal sequence SNTSESF (also known as AUR-7) of the previously described AUNP-12 peptide shown to inhibit PD-1 function [17]. While the full-length branched peptide results in optimal efficacy, the minimal sequence SNTSESF was shown to have surprisingly high activities [26]. Conjugation and multivalent display of SNTSESF on CPMV-AUNP resulted in moderately enhanced efficacy vs. CPMV alone. No improvement in efficacy was observed when CPMV was mixed with free peptide. The fact that free peptide showed no efficacy and conferred no improvement when mixed with CPMV may be explained by the dose and administration schedule. Here, we based dose and administration schedule on the typical CPMV schedule: weekly treatment using 100 µg CPMV; for every 100 µg CPMV-AUNP only 1 µg SNTSESF peptide was delivered. This low dose combined with weekly administration may not be sufficient to achieve efficacy of the anti-PD-1 peptide alone; however, data indicate that improved efficacy can be achieved through multivalent presentation and delivery by CPMV. It is clear that there is room for formulation improvement: ~25 peptide per CPMV were presented using a two-step bioconjugation protocol that utilizes a cysteine-terminated peptide and SM-PEG linker. CPMV offers 300 addressable surface lysine side chains and increased molar excess of peptide, and linker may yield increased bioconjugation efficiency, as an alternative one may consider orthogonal reactions such as Cu(I)-catalyzed azide-alkyne cycloadditions [27,28] or hydrazone-based coupling strategies [29]. Future studies will also concentrate on determining the most effective dose and administration schedule for CPMV-AUNP. Nevertheless, the fact that a suboptimal formulation yields increased efficacy is promising. The increased tumor residence time of the larger nanoparticle vs. the low molecular weight free peptide likely explains the observed efficacy. While the free peptide likely experiences rapid tumor wash out effects and proteolytic degradation, the nanoparticle conjugate offers stability. Similar phenomena were reported for other immunotherapy strategies; for example, CpGs which act as TLR-9 agonists, show increased potency when delivered by a nanoparticle, and this has been attributed to prolonged tumor residence and altered intratumoral distribution [30].

Mammalian in situ vaccination approaches and oncolytic viral therapies, e.g., Imlygic (Amgen) [6], have already made headways in the clinic; however, plant viral nanoparticles offer advantages compared to mammalian vectors or synthetic nanoparticle technologies: (1) Production through farming in plants is highly scalable for commercialization and the plant viruses can be stably stored (and are stable without cold chain requirements). (2) Plant viruses do not infect or replicate in mammalian cells, thus adding another layer of safety compared to oncolytic viral therapies. (3) The materials are uniform and monodisperse, a level of quality control and assurance is difficult to achieve using synthetic approaches. (4) Lastly, CPMV cancer immunotherapy is conceptually distinct from oncolytic cancer therapy: oncolytic viruses (including TVEC) function by targeting and killing cancer cells; however, CPMV targets innate immune cells to prime systemic anti-immunity (adaptive arm). A particular advantage is—because CPMV targets the innate immune system—presence of carrier-specific antibodies (which may be formed during repeat treatment schedules) are not neutralizing; rather, presence of anti-CPMV antibodies enhances the potency of the CPMV in situ vaccine over time. CPMV does not target cancer cells, but immune cells. Therefore, the presence of antibodies against CPMV opsonizes the nanoparticle, enhancing its uptake in innate immune cells, thus boosting the anti-tumor response [31].

Numerous combinatorial strategies of nanoparticles and immune checkpoint therapy have been reported and are in various stages of development; this includes the delivery of therapeutic antibodies as well as siRNAs to impede checkpoint receptor expression; we refer the reader to a recent review [32]. It is clear that there are many avenues to be pursued and the CPMV-AUNP nanoparticle, with further optimization, i.e., increased peptide loading, can be a powerful agent for cancer immunotherapy.

## 4. Materials and Methods

Production of Cowpea mosaic virus (CPMV): CPMV was propagated in *Vigna unguiculata* plants (Burpee’s Black-eyed pea No. 5, Burpee, Warminster, PA, USA) and purified from infected leaves using previously described methods [33]. CPMV preparations were stored in 0.1 M potassium phosphate (KP) buffer pH 7.0 at proteins concentrations <10 mg/mL and 4 °C. CPMV concentration was determined by UV/vis spectroscopy using a Nanodrop instrument and the CPMV specific extinction coefficient ε_260 nm_ = 8.1 mg^−1^ mL cm^−1^. The 260:280 nm ratio was also determined, and intact CPMV had a 260:280 nm ratio of 1.8. 

Synthesis of CPMVCPMV in 0.1 M KP buffer pH 7.-AUNP: CPMV-AUNP was obtained by conjugating AUR-7 peptide [17], SNTSESFGSGGGSGGC, to CPMV using a two-step bioconjugation protocol. SNTSESFGSGGGSGGC was obtained from Genscript (Piscataway, NJ, USA) with SNTSESF being the active region with reported antagonist activity of the PD-1 pathway [17]; GSGGGSGG was added as an intervening linker and the carboxy-terminal cysteine residue acted as ligation handle for conjugation to CPMV. First, CPMV was functionalized using a bi-functional *N*-hydroxysuccinimide-PEG_8_-maleimide (SM-PEG_8_) linker (Thermo Fisher Scientific, Waltham, MA, USA). It was then reacted with 500 M excess of SM-PEG_8_ linker at room temperature with constant mixing for 2 h at a 1 mg/mL. Second, SNTSESFGSGGGSGGC was added at 500, 1000 or 2000 M excess to CPMV. The resulting CPMV-AUNP conjugate was purified using 100 kDa molecular weight cut-off Amicon spin filters (Millipore, Sigma, Burlington, MA, USA). The product was resuspended in 0.1 M KP buffer pH 7.0 and stored at 4 °C.

Native and denaturing gel electrophoresis: CPMV, CPMV-SMPEG and CPMV-AUNP (10 μg per lane) were analyzed using 1% (*w*/*v*) agarose gel electrophoresis in 0.1 M TAE buffer (pH 6.5). Gels were stained with GelRed to stain the encapsulated RNA and Coomassie Brilliant Blue to stain the protein capsid. Denatured protein subunits (~10 μg per lane) were analyzed by polyacrylamide gel electrophoresis using 4–12% NuPAGE gels in 1× MOPS buffer (Invitrogen/Thermo Fisher Scientific, Carlsbad, CA, USA). Samples were denatured by boiling in SDS loading dye (Invitrogen/Thermo Fisher Scientific, Carlsbad, CA, USA) for 10 min. Gels were photographed under UV (when stained with GelRed) or white light (when stained with Coomassie Brilliant Blue) using an AlphaImager system (ProteinSimple, Santa Clara, CA, USA).

Transmission electron microscopy (TEM): CPMV and CPMV-AUNP (10 µL of 0.1 mg/mL) were deposited onto Formvar carbon-coated copper grids (Electron Microscopy Sciences, Hatfield, PA, USA) for 2 min at room temperature. The grids were then washed twice with deionized water for 45 s and stained twice with 2% (*w*/*v*) uranyl acetate in deionized water for another 30 s. A Tecnai Spirit G2 transmission electron microscope was used to analyze the samples at 80 kV.

Size exclusion chromatography (SEC): CPMV and CPMV-AUNP (100 µL of 1 mg/mL) were loaded onto a Superose-6 increase column on the ÄKTA Explorer system (GE Healthcare/Cytiva, Marlborough, MA, USA). The column was analyzed using a flow rate of 0.5 mL/min in KP buffer pH 7.0. 

CPMV and CPMV-AUNP therapy in a tumor mouse model: All animal studies were conducted upon approval and in accordance with the University of California, San Diego Institutional Animal Care and Use Committee (IACUC) guidelines. Six-week-old female C57BL/6J mice were purchased from The Jackson Laboratory. ID8-vegfA-defb29 murine ovarian serous carcinoma cell line [19] was cultured at 37 °C in RPMI 1640 complete media (Sigma Aldrich) supplemented with 10% (*v/v*) fetal bovine serum (Altanta Biologicals/R&D Systems, Flowery Branch, GA, USA), 1 mmol/L sodium pyruvate (MilliporeSigma, Carlsbad, CA, USA), 1% (*v/v*) penicillin/streptomycin mixture (MilliporeSigma, Carlsbad, CA, USA ), and 2 mmol/L L-glutamine (MilliporeSigma, Carlsbad, CA, USA). Cells were harvested and washed with RPMI medium. Eight-week-old mice were challenged with 2 × 10^6^ tumor cells in 400 µL sterile 1× PBS intraperitoneally (IP) on day 0. Mice were then randomly assigned into treatment groups and treatment was given by IP administration on day 8, 15, 22, 29, 36, and 42. The following treatment arms were assigned (200 µL IP injection): PBS control (*n* = 5), 1 µg AUNP (*n* = 3), 100 µg CPMV (*n* = 5), 100 µg CPMV-AUNP (*n* = 7), 100 µg CPMV+ 1 µg AUNP physical mixture (*n* = 7). Mice were weighed regularly to monitor ascites formation and measure tumor burden. Mice were euthanized with carbon dioxide when they reached the humane endpoint of 35 g of weight, indicating significant ascites formation. Tumor burden was measured by increase in body weight and data were analyzed and plotted using Graphpad Prism (version 9) software. 

## Data Availability

Data are available from the authors upon request.
